# Inhibitory Influence of *Enterococcus faecium* on the Propagation of Swine Influenza A Virus *In Vitro*

**DOI:** 10.1371/journal.pone.0053043

**Published:** 2013-01-07

**Authors:** Zhenya Wang, Weidong Chai, Michael Burwinkel, Sven Twardziok, Paul Wrede, Christiane Palissa, Bettina Esch, Michael F. G. Schmidt

**Affiliations:** 1 Institute of Immunology, Freie Universität Berlin, Berlin, Germany; 2 Molecular Biology and Bioinformatics, Charité - Universitätsmedizin Berlin, Berlin, Germany; Whitehead Institute, United States of America

## Abstract

The control of infectious diseases such as swine influenza viruses (SwIV) plays an important role in food production both from the animal health and from the public health point of view. Probiotic microorganisms and other health improving food supplements have been given increasing attention in recent years, but, no information on the effects of probiotics on swine influenza virus is available. Here we address this question by assessing the inhibitory potential of the probiotic *Enterococcus faecium* NCIMB 10415 *(E. faecium)* on the replication of two porcine strains of influenza virus (H1N1 and H3N2 strain) in a continuous porcine macrophage cell line (3D4/21) and in MDBK cells. Cell cultures were treated with *E. faecium* at the non-toxic concentration of 1×10^6^ CFU/ml in growth medium for 60 to 90 min before, during and after SwIV infection. After further incubation of cultures in probiotic-free growth medium, cell viability and virus propagation were determined at 48 h or 96 h post infection. The results obtained reveal an almost complete recovery of viability of SwIV infected cells and an inhibition of virus multiplication by up to four log units in the *E. faecium* treated cells. In both 3D4/21- and MDBK-cells a 60 min treatment with *E. faecium* stimulated nitric oxide (NO) release which is in line with published evidence for an antiviral function of NO. Furthermore, *E. faecium* caused a modified cellular expression of selected mediators of defence in 3D4-cells: while the expression of TNF-α, TLR-3 and IL-6 were decreased in the SwIV-infected and probiotic treated cells, IL-10 was found to be increased. Since we obtained experimental evidence for the direct adsorptive trapping of SwIV through *E. faecium*, this probiotic microorganism inhibits influenza viruses by at least two mechanisms, direct physical interaction and strengthening of innate defence at the cellular level.

## Introduction

Swine influenza is an infectious disease caused by RNA viruses which are highly contagious. Multiple strains of this virus are common throughout pig populations worldwide and cause significant economic losses in industry. At least three SwIV subtypes H1N1, H1N2 and H3N2 are currently circulating in the swine population despite regular vaccinations, and exchange of influenza viruses between human and swine is common and not a one-way street [1], [2]. The traditional vaccine is less effective because it cannot possibly include all the strains actively infecting people in the world. Therefore, therapeutic alternatives for preventing infections and maintaining the health of livestock are highly warranted.

Probiotics are defined as live microorganisms which when administered in adequate amounts confer a health benefit on the “host” (FAO/WHO, 2001). Although targeting the intestinal tract probiotics can also affect mucosal defence in general, including immune responses in the respiratory tissues [3], [4]. Probiotic bacteria, as a part of gut microflora, are reported to promote the host defense and to modulate the immune system [5]. Probiotics have been recently shown to mediate antiviral effects against certain viruses *in vitro* and *in vivo* [6], [7], [8], [9] and the effect of various strains of probiotics on the course of virus infections in pigs is being studied intensively. However, while some descriptive information on the effect of probiotics on model viruses such as vesicular stomatitis virus (VSV), transmissible gastroenteritis virus (TGEV) and rotaviruses [6], [7], [8], [9] are available, no such data are yet available for swine influenza viruses which are most important in view of their exquisite zoonotic capacity. It is commonly believed that the mammalian influenza viruses are restricted to the respiratory tissue and thus may hardly be affected by probiotics acting in the intestine. However, a recent report on the pathogenesis of seasonal influenza virus H1N1 in ferrets shows that this virus is also present in the intestine [10]. Furthermore it is world acknowledged that avian influenza viruses frequently infect in the intestine of the avian host [2]. Therefore it appears justified to include influenza viruses in studies on the probiotic inhibition of virus multiplication both *in vitro* and *in vivo*.

*Enterococcus faecium* NCIMB 10415 is authorized in the EU for safe use as a probiotic feed additive and therefore represents a suitable probiotic to study its possible anti-viral properties. We had previously carried out experiments with this probiotic in the context of bacterial infection which showed that *E. faecium* modulates intestinal immunity in piglets [11], [12]. In the present study we explored if *E. faecium* affects the replication of swine influenza virus H1N1 and H3N2 in a macrophage (3D4/21) and epithelial cell line (MDBK).

## Results

### Assessing the effect of *E. faecium* on the viability of 3D4/21 and MDBK cells

**Figure 1:**
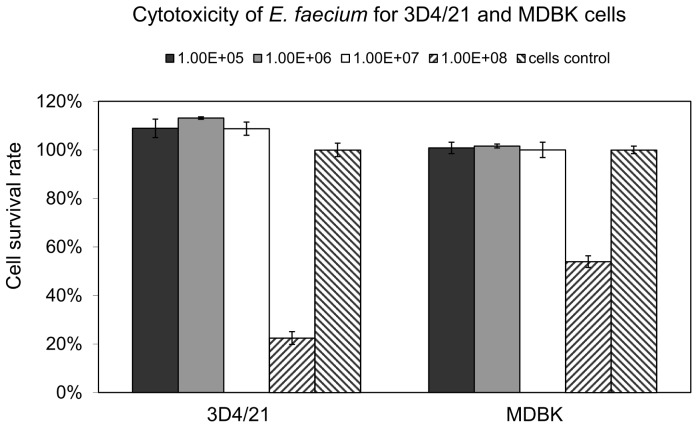
Cytotoxicity of *E. faecium* for 3D4/21 and MDBK cells. Different concentrations of *E. faecium* (1.00E+05, 1.00E+06, 1.00E+07, 1.00E+08 CFU/ml) were added to subconfluent 3D4/21 and MDBK cell monolayers and cell viability was assessed by an MTT assay after a 72 h exposure. Cell survival rates are given as relative values taking non-treated cells as 100%. The means ± standard deviations from three independent experiments are shown.

Cytotoxicity of *E. faecium* on both 3D4/21 and MDBK cells is shown in Fig. 1. Compared to control cells (100% cell survival rate), the application of *E. faecium* on the examined cell lines did not lead to any detrimental effects on cell integrity or metabolism unless the concentration exceeded the concentration of 1×10^7^ CFU/ml. As seen from the results compiled in Fig. 1, *E. faecium* at a concentration of 1×10^8^ CFU/ml had a severe cytotoxic effect especially for the macrophage cell line 3D4/21. Under the same conditions a proportion of about 60% of the MDBK-cells still survived in the presence of this probiotic. Based on these results, 1×10^6^ CFU/ml of *E. faecium* was applied for the interference studies described below.

### Effect of *E. faecium* on SwIV infection as detected by changes in cell viability

**Figure 2:**
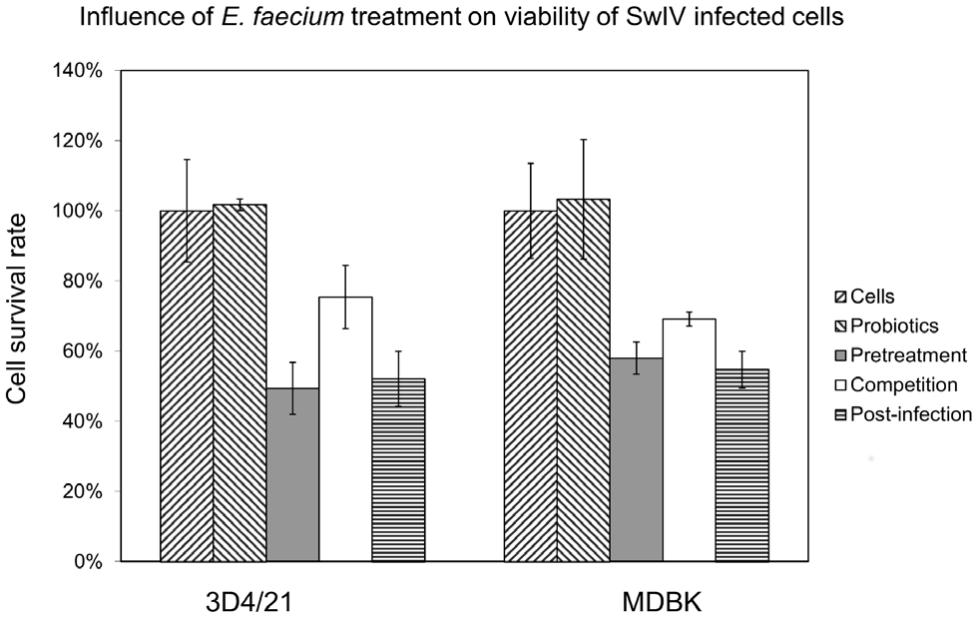
Cell viability of 3D4/21- and MDBK-cells treatment with *E. faecium.* Results are expressed as percent cell survival rates where non-treated and non-infected cells (first bar) served as controls (set at 100% survival rate) and SwIV-infected cells without *E. faecium* treatment as the complete damage marker (set at 0% survival rate). Virus infected cells with *E. faecium* treatment according to the modalities described in Fig. 6 are shown in last three columns of each group. Results represent means ± standard deviations from three independent experiments.

As expected from the above cytotoxicity study 1×10^6^ CFU/ml of *E. faecium* did not affect the viability of uninfected 3D4/21 and MDBK cells (Fig. 2, first two bars for each of the cell types). While SwIV at 48 or 96 hpi had destroyed the cell monolayers completely (defined as 0% survival, not shown) each of the treatment modalities with the above concentration of *E. faecium* resulted in a protection of the cells from SwIV infection. Among these the setup “competition”, where the probiotic bacteria and SwIV-inoculum are added to the monolayers together for 60 min, resulted in an 80% protective effect for 3D4/21 and in a 70% protective effect on MDBK cells. But even a pretreatment of the cells with *E. faecium* and the addition of the probiotic after completion of SwIV-infection both resulted in a significant rescue of the cells from “death” through the SwIV-infection (grey and horizontally marked bars in Fig. 2). These results were confirmed by another viability assay in which PI staining of dead cells is measured by flow cytometry (data not shown).

### Virus titer reduction in cells treated with *E. faecium*

**Figure 3:**
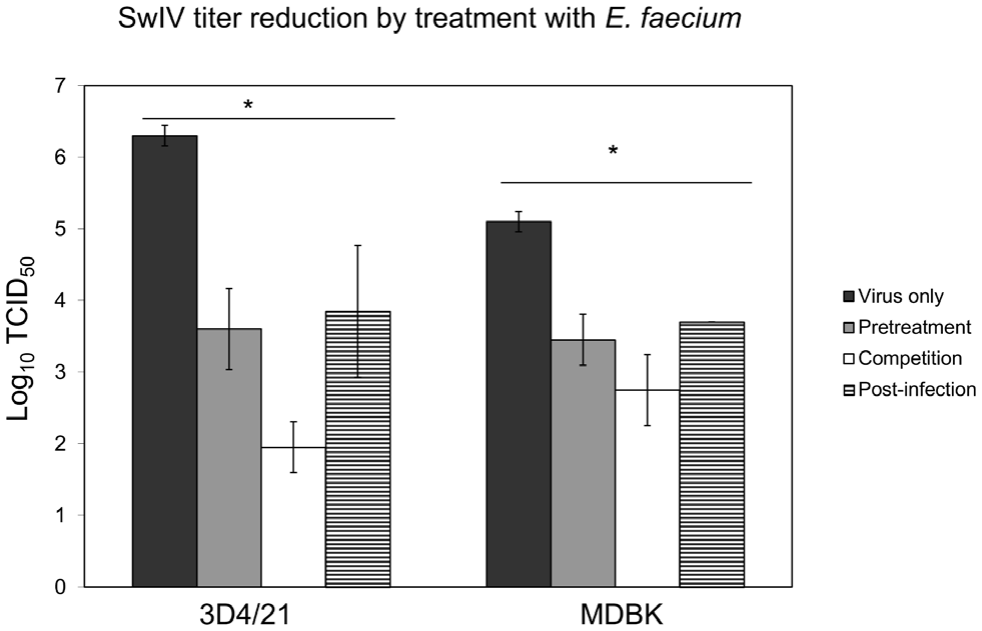
Influence of *E. faecium* on virus production in SwIV infected cells. 10^6^ CFU/ml *E. faecium* were added for 60 or 90 min to cells in 96-well plates according to the experimental design described in Fig. 6. Infection with SwIV was done at a MOI of 0.01. At 48 or 96 hpi, the supernatants were collected and virus titers determined by TCID_50_. Results are means ± standard deviations from three independent experiments. *P<0.05.

The effect of *E. faecium* treatment on viral titers was validated by the TCID_50_ assay. As shown in Fig. 3, the virus titer was decreased significantly after treatment of both types of host cells with *E. faecium*, but the degree of inhibition differed depending on whether the probiotic was present before, during or after infection with SwIV. An up to 4 Log_10_ TCID_50_ reduction was obtained when *E. faecium* and SwIV were present on the monolayers simultaneously indicating that direct competition between SwIV and the probiotic for presently unknown entities results in the most effective inhibition of virus production (see below) during the 48 h period before SwIV was quantified in the growth medium. These results are in line with those from the cell viability assay of SwIV-infected cells treated or non-treated with the probiotic shown in Fig. 2. Qualitatively the probiotic induced inhibition of SwIV appears to be the same with both types of host cells, but it appears to be somewhat more effective in the macrophage line 3D4/21. However, this could also be due to the lower SwIV-titers reached in the non-treated MDBK-cells which was about one log-unit less than in the non-treated 3D4/21-cells.

### *E. faecium* increases the production of NO

**Figure 4:**
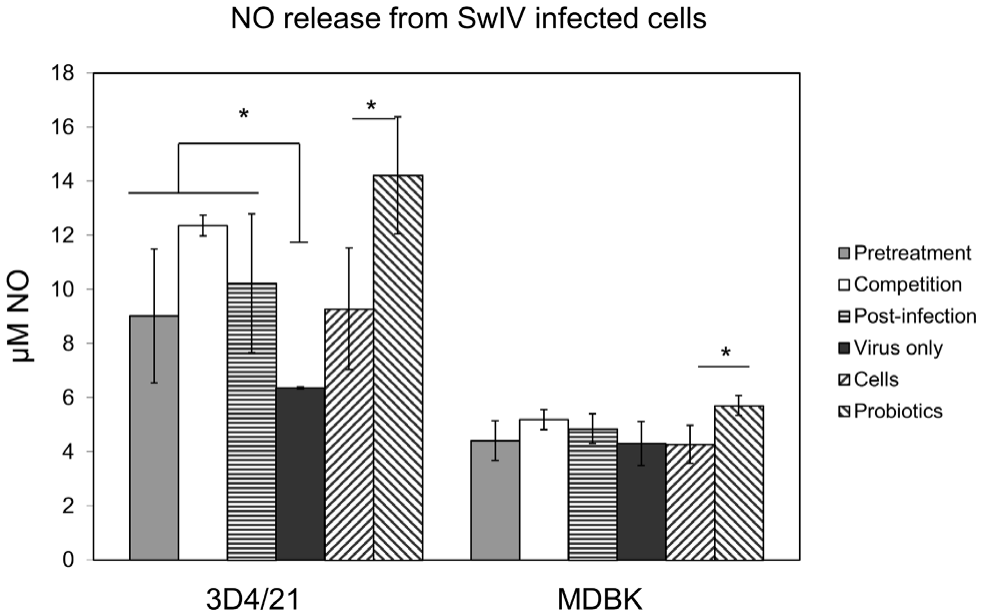
Effect of *E. faecium* on the nitric oxide (NO) release from 3D4/21 and MDBK cells. Released NO in the supernatant was measured by Griess assay according to the modalities described in Fig. 6. Cells only and cells treated with *E. faecium* are shown in last two columns of each group. Results are means ± standard deviations from three independent experiments. *P<0.05.

It is known from the literature that, beside multiple other functions, nitric oxide (NO) is an important physiological messenger and effector molecule for antiviral effects. Assessment of the secretion of NO under the influence of *E. faecium* revealed a most significant stimulating effect for 3D4/21 cells. As shown in Fig. 4, *E. faecium* increased the production of NO in both non-infected (bar on right side) and SwIV-infected cells (first three bars). As with the results shown above, the strongest stimulation was reached by *E. faecium* added to the host cells simultaneously with the virus (“competition”, white bar). In MDBK-cells stimulation on NO was much less pronounced. However the results shown in Fig. 4 indicate the same tendency as for 3D4/21-cells and are significant for the probiotic induced stimulation of NO in non-infected cells [13].

### Virus adsorption by *E. faecium*

It is possible that influenza virus particles could be engaged in direct physical interaction with the probiotic bacteria which may lead to a loss of infectivity. To address this question we included an experiment where virus particles were mixed with probiotic bacteria in a test tube and incubated for 1.5 h at room temperature (“preincubation”). After low speed centrifugation of the mixture to sediment *E. faecium*, the bacterium-free supernatants were titrated for infectious SwIV in a TCID_50_ assay. As seen from the data in Table 1, virus titers were reduced by about two log-units in both types of host cells. If virus was bound to bacterial cells during the preincubation period, it should be present in the bacterial sediment after the centrifugation step. To probe this possibility, the bacterial sediments were subjected to real time-quantitative PCR in which primers for SwIV M-protein were applied. This resulted in the finding that up to 50% of the input virus was detectable in the bacterial sediment (data not shown). As shown in Table 1, SwIV in the supernatants from low speed centrifugation had lost infectivity significantly both on 3D4/21- and MDBK-cells. However, when NO was measured in the same cultures, an increase was recorded for both cell types.

**Table 1:** Loss of infectivity by direct physical interaction of SwIV and *E. faecium*.

Cell line	3D4/21		MDBK	
	Virus control	Virus+ *E. faecium*	Virus control	Virus+ *E. faecium*
**Log_10_ TCID_50_**	6.27±0.12	3.63±0.15**	5.47±0.64	3.60±0.53**
**µm NO**	6.36±0.03	10.82±1.72**	4.28±0.70	5.21±0.41

After preincubation of SwIV and *E. faecium* for 1.5 h, the mixture was centrifuged and supernatants were transferred onto monolayers of 3D4/21 and MDBK cells to determine the virus titers in the original supernatants by TCID_50_ at 48 hpi or 96 hpi. Aliquots from the growth medium of the virus titration were taken to determine NO release. As a control, SwIV was preincubated without adding any *E. faecium* and the samples processed in parallel to the ones with the probiotic. Results are means ± standard deviations from three independent experiments.

** P<0.01.

### Cytokine expression in SwIV-infected cells under the influence of *E. faecium*

**Figure 5:**
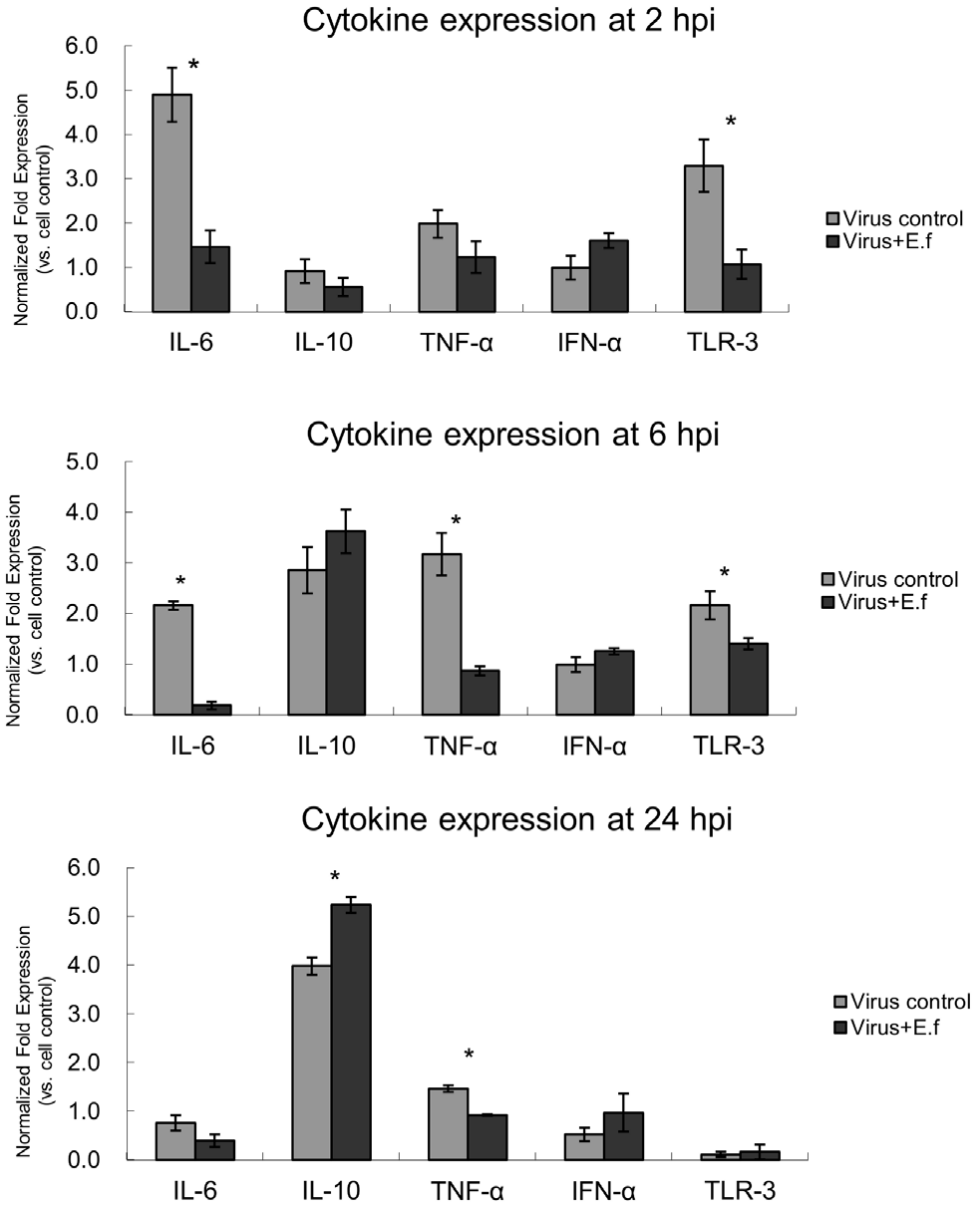
Cytokine expression at 2 h, 6 h and 24 h. Cytokine response of 3D4/21 cells to SwIV challenge was determined after a 1 h treatment with 10^6^ CFU/ml *E. faecium* during the infection period (Competition assay, see Fig. 6). Selected cytokines (IL-6, IL-10, TNF-a, IFN- α and TLR-3) were measured at 2, 6 and 24 hpi. Results are means ± standard deviations from three independent experiments. *P<0.05.

The observed inhibition of virus multiplication and stimulation of NO in the experiments where the probiotic was added to the cells before SwIV was in play or after the virus had entered the cells (Fig. 1, Fig. 3) indicate, that *E. faecium* may influence cellular factors which affect virus growth. We therefore analysed the 3D4/21 cell expression of cytokines (IL-6, IL-10, TNF-α, IFN- α) and TLR3 which are known for their potential to modulate virus production. The results from quantitative RT-PCR shown in Fig. 5 reveal a decreased expression of these mediators when compared to the non-treated samples (SwIV-infected 3D4/21-cells without *E. faecium*). On the other hand, the immunosuppressive cytokine IL-10 showed a low expression at 2 h, but increased strongly at 6 h and 24 h in the probiotic-treated cultures (Fig. 5). Furthermore, *E. faecium* promoted an increased expression of IFN-α, at 2 h, 6 h and 24 h post SwIV infection although without significance of the values for the virus group and *E. faecium* treated group, so this effect can only be regarded as a tendency.

## Discussion

Probiotic microorganisms have been mainly studied in the context of bacterial infections of the gastrointestinal tract which is the natural target tissue of probiotics. However, there are a few reports which indicate that upon oral intake, probiotics can also affect infections of the respiratory tract [14]. There are also reports in the literature where probiotics induce antiviral activity *in vitro* and are even applied as a medical treatment against persistent virus infections in humans and animals.

We chose the zoonotic swine influenza viruses as a novel study object to test for the antiviral potential of the probiotic *E. faecium* and to elucidate its mechanisms of action and we present the results from *in vitro* experiments using porcine H1N1- and H3N2-influenza virus in MDBK- and 3D4/21 cells, respectively. Our results demonstrate that the probiotic *E. faecium* effectively protects host cells from swine influenza virus infection and are in support of the above author's hypothesis, that probiotics are not only useful to inhibit enteric viruses, but may also have potential for the control of respiratory viruses.

**Figure 6:**
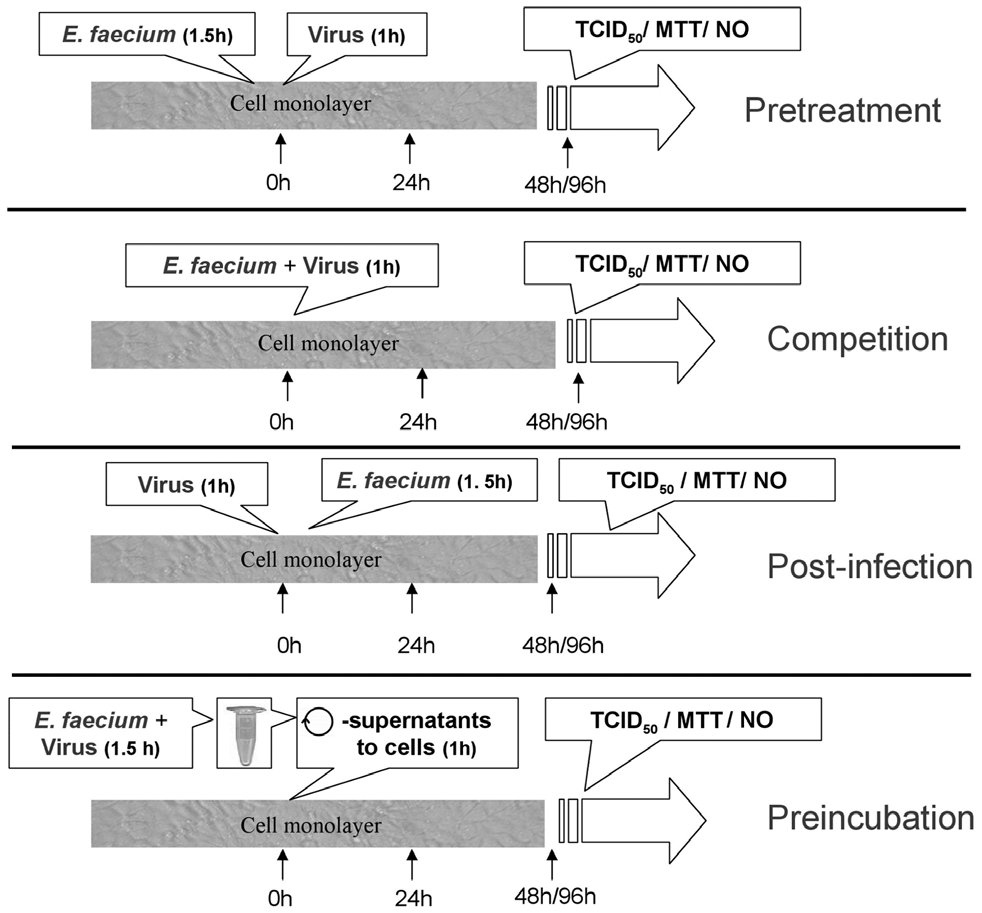
Experimental design of dose response study of probiotic effect on SwIV. (1) Pretreatment of cell monolayers with probiotics for 1.5 h before SwIV infection (Pretreatment). (2) Probiotics and virus were added together to the cells (Competition). (3) Treatment of cell monolayer with probiotics 1 h after SwIV infection (Post-infection). (4) After preincubation of SwIV with probiotic bacteria, the mixed samples were centrifuged and the supernatants were added to the cells (Preincubation).

Two different SwIV strains were chosen which are currently circulating in the pig population, H1N1 (A/Swine/Greven/IDT2889/2004) and H3N2 (A/Swine/Bondelum/IDT5959/2007). As an established model for the present *in vitro* study an epithelial- (MDBK-cells) and a porcine alveolar macrophage cell line (3D4/21-cells) were utilized. It can be argued that the concentration of the probiotic utilized may not reflect the situation in the target tissue *in vivo*. However, the concentration chosen for treatment of the cell cultures (10^6^ CFU/ml) reflects the same concentration which was determined in the gut of piglets fed *E. faecium* as a supplement during previous feeding trials in our research consortium [15]. In order to find out during which period of the SwIV replication cycle the probiotic has the most stringent effect, *E. faecium* was added for a brief period of time (60 or 90 min) to the host cells either before, during or after virus infection (Fig. 6). The results indicate that the simultaneous addition of virus and *E. faecium* to the host cell monolayer was the most effective timing for the inhibition of virus multiplication. As shown in Fig. 3 and Fig. 4, this experimental setup (termed “competition”) resulted in a 4 log-unit reduction of virus titer and in a concomitant rescue of cell viability. Since a 1 h exposure of the monolayers to *E. faecium* before SwIV-infection and a 1 h treatment after completion of virus infection also led to a 2–3 log-unit loss of virus titer, the probiotic must alter host cell factors which apparently results in an inhibition of influenza virus multiplication. Obvious candidates for such factors are mediators of cellular defence processes.

The expression of NO and its subsequent increased activity has previously been reported to play a role in the host response to multiple viral families, and in various host species [16], [17]. In addition to its antiviral properties, NO has been described to modulate intestinal barrier function, gut motility, iron transport, and has been implicated in numerous infections and non-infectious diseases of the intestine [18]. We found that *E. faecium* increased the expression of NO in both 3D4/21 and MDBK cells (Fig. 5). All the samples collected after treatment with *E. faecium* showed significantly increased NO-values when compared to the non-treated counterparts on 3D4/21 cell line. This is consistent with the hypothesis that high NO levels are associated with decreased virus production.

In addition to stimulating NO-release, probiotics could also affect the expression of cytokines and other immune mediators relevant for the innate immune response to viral infections. We therefore determined the expression of selected mediators in SwIV-infected host cells (3D4/21-cells only). As seen from Fig. 5, *E. faecium* promoted an increased expression of IFN-α. Since the difference between the values of non-treated and *E. faecium* treated cells were found to be non-significant, IFN-α can be ruled out as the main immunoregulatory cytokine that could lead to *E. faecium* induced inhibition of SwIV-infection. Another cytokine stimulated by the probiotic treatment was IL-10, which is a typical Th2 cytokine that is initially repressed in virus infected cells but then expressed at higher levels later in infection to control the initial inflammatory response to infection. Interestingly, this cytokine is clearly enhanced in the macrophage cell line upon *E. faecium* treatment and thus could support cellular control of SwIV infection. Two pro-inflammatory cytokines were found to be clearly reduced in the SwIV-infected 3D4/21-cells treated with the probiotic, IL-6 and TNF-α. Secretion of IL-6 by macrophages is known to play an indirect immunoregulatory role in the immune response to viral infection [19], and TNF-α acts as an inflammatory cytokine by triggering a cascade of cytokine production [20] Since both IL-6 and TNF-α are downregulated by *E. faecium* in SwIV-infected 3D4/21 cells, the reduced inflammatory response caused by some cytokines at the cellular level may contribute to the antiviral effect of the probiotic. Toll-like receptor 3 (TLR-3) was the first identified antiviral TLR to have a central role in the host response to viruses [21]. Our experimental data show that the treatment of SwIV-infected 3D4/21-cells with *E. faecium* led to an decreased expression of TLR-3 at 2 h and 6 h post infections compare to virus alone which suggest that the probiotic induced modulation of this receptor may have a role in the antiviral function of *E. faecium*. Since *E. faecium* acts most inhibitory when it is added together with the virus particles, we assessed whether SwIV might be physically trapped or inactivated by the probiotic bacteria in a mixed incubation as detailed as “preincubation assay” in the experimental design shown in Fig. 6 (lower panel). The results summarized in Table 1 show that a substantial portion of the input virus particles are indeed trapped by the bacteria since virus infectivity is lost from the supernatants and viral genome equivalents are detected in the bacterial sediments after low speed centrifugation of the incubation mixture (data not shown). Thus under such experimental conditions two antiviral functions of the probiotic may operate synergistically and add up to produce a more severe inhibition of SwIV.

The results presented altogether show that the probiotic *E. faecium* quite effectively inhibits the multiplication of swine influenza viruses in relevant cell culture systems. The antiviral mechanism of this probiotic is probably manifold since it was found to act on both the virus particles and the host cells. However, at least a few inhibitory parameters could be identified: *E. faecium* bacteria are able to adsorb SwIV-particles and to alert the cells by mediating a rapid antiviral response through modulating the expression of defence relevant mediators. Amongst these IL-6, TNF-α, IL-10, IFN-α and TLR-3 were identified as entities modulated by the probiotic treatment. It is realized that *E. faecium* can induce much more complex reactions in a treated tissue since only a few mediators could be assessed in this study. One common denominator of probiotic action could be NO which is a mediator affected by many cellular signaling cascades. In line with publications for other virus-host cell systems, our results point to a central role of NO which is stimulated upon the treatment with the probiotic and which may mount an improved cellular defence response against SwIV-infection in tissues which were stimulated with a probiotic.

Based on the *in vitro* data shown here for a porcine influenza virus, we hypothesize that the use of *E. faecium* as a probiotic feed (or food) additive has the potential of reducing influenza virus infections in mammalian tissues. SwIV challenge experiments with piglets which are fed *E. faecium* as a supplement are presently in progress in order to test this hypothesis.

## Materials and Methods

### Virus, cells and probiotic

The SwIV strains H1N1 (A/Swine/Greven/IDT2889/2004), H3N2 (A/Swine/Bondelum/IDT5959/2007), Madin-Darby Bovine Kidney (MDBK) cells [22] used in this study were a generous gift from Dr. R. Dürrwald (Impfstoffwerk Dessau-Thornau, Germany). Stock virus of H1N1 and of H3N2 was propagated in MDBK and in MDCK cells, respectively. The H1N1 strain was used on MDBK cells and the H3N2 strain was used on 3D4/21 macrophages. MDBK and MDCK cells were maintained in Dulbecco's modified Eagle's medium (DMEM; PAN Biotech) supplemented with 5% fetal calf serum (Hyclone), and 1% penicillin/streptomycin (Biochrom). The porcine continuous monomyeloid cell line 3D4/21 established from primary porcine alveolar macrophages [23] were kindly provided by Prof. A. Cencič (University of Maribor, Slovenia) and Dr. Hana Weingartl. 3D4/21 cells were maintained in Advanced Dulbecco's modified Eagle's medium (Gibco) supplemented with 10% fetal calf serum (Hyclone), and 1% penicillin/streptomycin (Biochrom). *E. faecium* NCIMB 10415 (Cylactin®, Cerbios-Pharma SA) was maintained in Todd-Hewitt broth (Roth). All experiments were done in triplicate.

### Cytotoxicity of *E. faecium*

To determine possible cytotoxic effects of *E. faecium*, different concentrations (1.00E+05, 1.00E+06, 1.00E+07 or 1.00E+08 colony forming units (CFU)/ml) were added to 3D4/21 and MDBK cell monolayers in 96-well plates (Greiner Bio-One) for 72 h and cell viability was monitored by a methylthiazolyl-diphenyl-tetrazolium bromide (MTT) viability assay. Briefly, cell monolayers were washed after the 72 h treatment period, 20 µl MTT in PBS was added to each well and the plates were further incubated at 37°C in a CO_2_ incubator for 1.5 h. Solubilisation of the formazan crystals formed during this period was achieved by the addition of DMSO solution. The absorbance (OD) at 570 nm was measured using a microplate reader (Tecan, Germany). Cell survival rate was determined as bacteria average OD value/control average OD value.

### Experimental design of interference experiments

For interference studies, infection of cells with both strains of SwIV was done at a multiplicity of infection (MOI) of 0.01. *E. faecium* was applied at a concentration of 10^6^ CFU/ml. In avoid of carry over effects, after *E. faecium* was washed off two times, 1% penicillin/streptomycin was used to stop the propagation of *E. faecium*. The schematic in Fig. 6 depicts our experimental setup for studying the interference between *E. faecium* with SwIV-infection in the two cell culture systems. It allows us to define at what time during virus growth the addition of the probiotic is most effective. The preincubation setup should reveal whether the probiotic bacteria have a direct effect on the virus particles without any involvement of host cells. The MTT assay was used to measure the mitochondrial function, which serves as a viability index of metabolically active cells. After the experimental incubation period, the MTT assay was applied as described above. The percentage of metabolically active cells treated with probiotic bacteria and the percentage of protection from cytopathic effect achieved was then calculated. All data represent the average values for a minimum of six wells of three independent experiments.

### Determination of viral titers

Virus titers were calculated as 50% tissue culture infectious doses (TCID_50_) by titrating supernatants containing H1N1 or H3N2 SwIV in tenfold steps on 3D4/21 cells or MDBK indicator cells, respectively. Three days after infection, indicator cells were stained by Giemsa (Sigma) and the cytopathic effect (CPE) was observed macroscopically and under the microscope. The results of all TCID_50_ assays were calculated according to the Reed and Muench method [24].

### Assessment of nitric oxide (NO) release

NO release was determined by measuring the amount of NO_2_^−^ released into the culture medium by use of the Griess-Assay (Promega) according to the manufacturer's instructions. Briefly, 50 µl of each experimental sample was transferred into a 96 well plate in triplicate. Defined standard samples were assessed in parallel to produce a standard curve. 50 µl of a sulfanilamide solution (1% sulfanilamide in 5% phosphoric acid) were added to each well for 10 minutes at room temperature. Then 50 µl of the NED Solution were dispensed at room temperature for 10 minutes and absorbance (OD) at 570 nm measured using a microplate reader (Tecan). NO release in each sample was calculated by use of the nitrite standard curve generated in parallel.

### Virus adsorption to *E. faecium* (preincubation assay)

To investigate if virus could be trapped by probiotics, *E. faecium* (1.00E+06 CFU/ml) were mixed with 0.01 MOI SwIV in a total of 1 ml DMEM for 90 min co-incubation at 37°C in a CO_2_ incubator for 1.5 h. The mixture was then centrifuged at 3,500 rpm for 10 min (compare Fig. 6 – Preincubation assay). Sediments were prepared for quantitative PCR and supernatants were used to infect cells. Total RNA was isolated from sediments and M protein of SwIV was amplified and compared to virus control. The virus titer and NO release assays mentioned above were carried out at 48 or 96 hpi.

### Real-time PCR for the expression of cytokines

After the various treatment periods, 3D4/21 cells were collected from the wells at 2 h, 6 h and 24 h. Total RNA was isolated from cell samples by use of the Gene MATRIX RNA Purification Kit (EURx). Reverse transcription (RT) was performed using the RevertAidTM First Strand cDNA Synthesis Kit (Fermentas) according to the manufacturer's instructions. PCR reactions were performed in a total volume of 25 µl in an iCycler iQ detection system (Bio-Rad Laboratories). The expression of each gene was analyzed using the relative quantification method [25]. The designations of genes, the primer sequences, the annealing temperatures, and the sizes of the amplification are listed in Table 2.

**Table 2:** PCR primers.

Gene	Primer pairs (5′- 3′)	Product (bp)	Accession number
**β-Actin**	Forward: CGGGACCTGACCGACTA	233	DQ845171.1
	Reverse: AAGGTCGGGAGGAAGGA		
**IL-6**	Forward: AACGCCTGGAAGAAGA	229	Ab194100
	Reverse: AACCCAGATTGGAAGC		
**IL-10**	Forward: GCATCCACTTCCCAACCA	446	EF433759
	Reverse: TCGGCATTACGTCTTCCAG		
**IFN-α**	Forward: GCTCCTGG ACA AATG	197	NM214393
	Reverse: GCTGCTGATCCAGTCC		
**TNF-α**	Forward: ACGCTCTTCTGCCTACTGC	388	NM214022
	Reverse: TGGGCGACGGGCTTATC		
**TLR-3**	Forward: AACCAGCAACACGACT	110	Ab111939
	Reverse: TTGGAA AGCCCATAA A		

### Statistical analysis

All calculations were performed with IBM SPSS 19. Data analysis for virus titers and NO release were performed by two factorial ANOVA followed by a post hoc test (Scheffe). Data analysis for cytokine expression was performed by paired, two tailed t-test. P values of <0.05 were considered statistically significant. P values of <0.01 were considered statistically very significant. All data are given as the mean ± standard deviation.
